# Engaging the Public to Identify Opportunities to Improve Critical Care: A Qualitative Analysis of an Open Community Forum

**DOI:** 10.1371/journal.pone.0143088

**Published:** 2015-11-18

**Authors:** Melissa L. Potestio, Jamie M. Boyd, Sean M. Bagshaw, Daren Heyland, Peter Oxland, Christopher J. Doig, Dave Zygun, Henry T. Stelfox

**Affiliations:** 1 Research Priorities & Implementation, Alberta Health Services, Calgary, Canada; 2 Department of Community Health Sciences, University of Calgary, Calgary, Canada; 3 W21C Research and Innovation Centre, University of Calgary, Calgary, Canada; 4 Division of Critical Care Medicine, Faculty of Medicine and Dentistry, University of Alberta, Alberta Health Services, Edmonton, Canada; 5 Department of Critical Care Medicine, Queen’s University, Clinical Evaluation Research Unit, Kingston General Hospital, Kingston, Canada; 6 Family Advisor, Critical Care, Alberta Health Services, Calgary, Canada; 7 Patient and Community Engagement Researcher (PaCER), University of Calgary, Calgary, Canada; 8 Department of Critical Care Medicine, University of Calgary, Alberta Health Services, Calgary, Canada; University of Tübingen, GERMANY

## Abstract

**Objective:**

To engage the public to understand how to improve the care of critically ill patients.

**Design:**

A qualitative content analysis of an open community forum (Café Scientifique).

**Setting:**

Public venue in Calgary, Alberta, Canada.

**Participants:**

Members of the general public including patients, families of patients, health care providers, and members of the community at large.

**Methods:**

A panel of researchers, decision-makers, and a family member led a Café Scientifique, an informal dialogue between the populace and experts, over three-hours to engage the public to understand how to improve the care of critically ill patients. Conventional qualitative content analysis was used to analyze the data. The inductive analysis occurred in three phases: coding, categorizing, and developing themes.

**Results:**

Thirty-eight members of the public (former ICU patients, family members of patients, providers, community members) attended. Participants focused the discussion and provided concrete suggestions for improvement around communication (family as surrogate voice, timing of conversations, decision tools) and provider well-being and engagement, as opposed to medical interventions in critical care.

**Conclusions:**

Café participants believe patient and family centered care is important to ensure high-quality care in the ICU. A Café Scientifique is a valuable forum to engage the public to contribute to priority setting areas for research in critical care, as well as a platform to share lived experience. Research stakeholders including health care organizations, governments, and funding organizations should provide more opportunities for the public to engage in meaningful conversations about how to best improve healthcare.

## Introduction

Each year, thousands of people are admitted to an intensive care unit (ICU) with life-threatening conditions. These patients include members of the public who were previously healthy and have become afflicted with life-threatening illnesses, patients with acute exacerbations of chronic medical problems and the frail elderly [1]. ICU care is resource intensive [2]. These patients have complex medical problems that require twice as many tests and treatments as other hospitalized patients [3], and leave them with limited physiological reserve to tolerate additional illness and a vulnerability to adverse events related to patient need/clinical care mismatches [4]. As such, there is urgency to optimize the quality of patient care in the ICU.

Efforts to improve health care risk being ineffective unless they reflect what patients and their family members need and want. The Institute of Medicine (IOM) defines quality as “the degree to which health care services for individuals and populations increase the likelihood of desired outcomes and are consistent with current professional knowledge [5].” Central to this definition is that desired outcomes be consistent with both clinical goals and patients’ own goals. In Crossing the Quality Chasm, the IOM emphasized the importance that care be patient-centered, “respectful of and responsive to individual patient preferences, needs, and values [6].” The Canadian Institutes of Health Research (CIHR) has similarly implemented a patient-oriented research strategy [7]. Little is currently known about the priorities of critically ill patients who epitomize the challenges of providing patient-centered acute care. As such, we used the CIHR Café Scientifique program to engage the public in an open forum to contribute their views on how to improve the care of critically ill patients in ICUs.

## Methods

A panel of researchers and decision-makers, with expertise in both healthcare innovations and in critical care, and a family representative led a Café Scientifique with the public in a three-hour event on Thursday June 5th, 2014 at a pub in Calgary, Canada. Café Scientifique is an international public science initiative designed to promote discussions around health related issues of interest to the general public, and in turn foster public engagement and make science accountable to the public [8]. We designed our Café Scientifique to use interactions (panel-public, public-public, panel-panel) to build upon individual comments, while encouraging the expression of unique thoughts, creating a synergy of ideas [9]. The objective was to engage the public to understand how to improve the care of critically ill patients. Thus the primary question asked to the Café participants was “What are the most important ways that you feel that we can improve the care of critically ill patients, those with life-threatening disease”?

We targeted the Café Scientifique to members of the general public including patients, families of patients, health care providers, and members of the community at large. Our marketing plan was multifaceted to attract a diverse representation of the public using announcements through, Alberta Health Services (www.albertahealthservices.ca), O’Brien Institute for Public Health, University of Calgary (www.iph.ucalgary.ca), EventBrite^R^, Facebook^R^, local media, and word-of-mouth.

The agenda for the three hour meeting was moderated by the medical director of the Critical Care Strategic Clinical Network (CCSCN) [http://www.albertahealthservices.ca/9437.asp] and included a brief presentation of current (activities in progress) and future (priorities identified through the network’s stakeholder engagement process) innovation activities and examples of how research can address innovation needs. Each panelist provided a 10 minute presentation on an example of research addressing an innovation priority, and the family member panelist provided insight into the experience of having a loved one in the ICU. These were followed by group discussion. The topics covered by the panelists spanned the four CIHR pillars (biomedical, clinical, health services; and population health research) and included the content areas of sepsis, fluid resuscitation, kidney injury, end of life care, and family member experience of ICU care.

We obtained written consent from all participants to audio-record the proceedings. Two researchers (MP, JB) also recorded qualitative observations and memos during the event. Participants were provided with a short survey to elicit feedback on the event (S1 Appendix). All data was collected anonymously (i.e., no personal identifiers). The project was approved by the University of Calgary Conjoint Health Research Ethics Board REB-13-1158.

The data from the Café Scientifique was translated and transcribed verbatim. Conventional qualitative content analysis was used to analyze the data [10, 11]. The analysis process involved an initial familiarization period involving immersion in the data by several sessions of reading the transcript [12]. Two researchers (MP, JB) independently reviewed the transcript to familiarize themselves with the data. The inductive analysis occurred in three phases: coding, categorizing, and developing themes [13, 14]. Consensus amongst the investigators resulted in a rich, detailed explanation of descriptors for coding each question with the coding scheme developed through data immersion and frequent conversations between the researchers [15]. Collaboration among researchers throughout the process ensured rigor of the codes and established in depth inter-coder validity [16]. As Lincoln and Guba (1985) suggest, coding and recoding were determined as complete when the analysis itself appeared to have run its course–when all of the incidents were readily classified, categories were “saturated”, and sufficient numbers of “regularities” emerged [17]. Codes were placed into broad categories that corresponded to the major unit of analysis (S2 Appendix). As categories emerged, their theoretical properties were defined. Comparisons between multiple categories were carried out in order to locate similarities and differences between them and finally to obtain a holistic view of the data, categories were synthesized into themes. The themes explained the text within the data, highlighting key factors that can be used to inform our understanding of participants’ suggestions for improving the care of critically ill patients.

Responses to the feedback survey were entered into a database and descriptive analysis was used to explore participant satisfaction with the event (S3 Appendix). Responses from the open-ended questions were included in the content analysis.

## Results

Thirty-eight members of the public representing former ICU patients, family members of patients, providers and interested community members attended the Café Scientifique and consented to participate. When participants were asked for suggestions on how to improve the care of critically ill patients in the ICU, responses did not focus on the actual medical interventions received or the need for new clinical innovations. When the medical care received was mentioned, patients were extremely satisfied.

“Like in 1997 I had fantastic care, obviously, or I wouldn’t be here today.”

“…the nurses and the doctors really did a good job of letting me know that you know mom was very, very well cared for.”

“And they tried to resuscitate her and she was on a breathing tube and that kind of thing. I think that just what was great about my experience with my mom is that the doctors, and the nurses especially, were just so, so beautiful and so, so caring and it was just really, really tough.”

“We were blessed with wonderful care at [hospital name] in that respect, nothing but good things to say.”

Rather, conversations focused around three themes: *Communication*, *Provider Well-Being*, and *Engagement*.

### Communication

Participants spoke about the importance of good communication between patients, family members, and providers and focused discussions around three sub-themes: *Family as Surrogate Voice*, *Timing of Conversations*, and *Decision Tools*. Participants recognized that communication within the ICU can be challenging due to the nature and severity of illness. As such, participants spoke about the importance of the family member as the surrogate voice of their loved one. In addition, participants expressed the importance of appropriate timing for conversations. Due to the difficult and stressful nature of ICU illness and concerns over how to effectively communicate the wishes of loved ones, participants expressed a desire for tools to support decision-making.

#### (1) Family as Surrogate Voice

Participants who were family members of ICU patients spoke about the important role they played as being the patient’s voice. Family members felt a lot of responsibility for ensuring their family member’s wishes were communicated to providers.

“At that time when I went to see her she had the tubes in her throat and she couldn’t speak so we kind of wrote back and forth on an envelope. And then she kept writing over and over again “I want to be resuscitated. Please make sure you talk to the doctor and tell the doctor I want to be resuscitated.” So I made sure that that’s exactly what I would do.”

#### (2) Timing of Conversations

In the ICU environment, family members often communicate on behalf of their loved ones. As such, family members must be confident in their loved ones’ values and wishes to be able to communicate this to providers. Many participants spoke about the importance of having conversations around values, wishes, and end-of-life care before someone becomes ill. Participants agreed that once a family member is admitted to an ICU it is often too late to find out their wishes, putting the family into an incredibly difficult position. That being said, participants also recognized the difficulty of having such conversations with family members.

“Way too late when people hit the ICU to be having these conversations. Way, way, way too late, first of all.”

“Just a few thoughts, I think it’s been interesting to hear that we agree that the conversation needs to happen earlier on the front line. We've heard it from the experts that the conversations that are happening in the ICU are too late and not effective and you’re putting together tools to help the frontline healthcare workers have that conversation. So I guess I’m wondering is why don’t we just get rid of the middleman? How do I get you as the death experts in the family doctor's office?”

“So if we do it in the ICU we’ve got it wrong. When that person is diagnosed with their end stage renal disease, with their congestive heart failure, with their COPD, with their neuromuscular disease, if it’s done months and years in advance it’s probably a far better time to be doing it than that new care provider at the time somebody needs admission to the ICU or is already in the ICU.”

“It’s too difficult and it’s too late when you’re in the ICU trying to make those decisions.”

#### (3) Decision Tools

Participants recognized that in the ICU environment, family members often have to make difficult decisions. As such, participants spoke about the need for decision tools to support families in making such decisions. Participants supported the development of decision tools to help people think through and clarify their values so they are better prepared to make difficult decisions.

“…we need to see the support tools to help people understand the decisions that need to be made at the end-of-life. Should I be resuscitated? Should I go to the ICU? What’s this mechanical ventilation thing all about?”

### Provider Well-Being

Participants expressed interest in the need to address the potential stress, fatigue, and burnout experienced by ICU providers. Participants were unsure how providers dealt with the emotional stress of working in the ICU environment and thus questioned if there was a need to increase opportunities for providers to engage in conversations around stress and suffering.

“We know that suffering happens in ICU, but we don’t often speak to it and so my question for the panel, and also for the audience, is how do we address suffering of our patients and how do we address the suffering of ourselves as healthcare providers caring for patients in often the worst times in their life?”

“But I do wonder about the healthcare providers because you know when your loved one dies and eventually they leave the room presumably another patient could come in with another illness and to what extent do the healthcare providers get together and talk about it? What kind of caring do they have? What kind of an opportunity, because they [go] through some grief too.”

### Engagement

Café participants felt strongly that engaging the public in conversations around care in the ICU is important to help guide innovation, so that it reflects what the public needs and wants. Participants expressed a desire to have more opportunities to be involved in discussions with healthcare providers and decision-makers. Participants recognized that historically engagement opportunities have been limited and thus expressed gratitude for being invited to the Café Scientifique. In particular, participants felt hosting public forums were the most appropriate way to engage the public in conversations.

“First of all, thank you for having this forum tonight, this is fantastic. I appreciate the opportunity to be able to come and to speak and to share my thoughts.”

“It’s like [name] said it's through storytelling and sharing that change happens. So thank you.”

“Just kudos to everybody here for actually getting involved in this and bringing it to the forefront. Very important.”

### Participant Feedback

The results of our quantitative feedback survey, response rate of 73.7%, indicated overwhelming satisfaction with the Café. Participants provided positive feedback (satisfied or very satisfied) on many aspects of the event including scheduling and timing of the event (85.7%), exploration of the event’s theme (85.7%) and relevance of the discussion (92.9%) and likelihood to attend a future event (92.9%), the venue (75.0%) and food and beverages provided (89.3%). Participants reported hearing about the Café Scientifique through various avenues (categories are not mutually exclusive). Most participants (64.3%) reported hearing about the Café Scientifique through email distribution lists, websites, and social media platforms. Other means of recruiting participants included poster advertisements (14.3%) and word of mouth (39.2%).

## Discussion

The Café was planned with the intention to engage the public in discussing how to improve the care of critically ill patients and solicit feedback on what innovations are needed and most relevant. However, despite three of the four panelists initiating discussions centered around the role of research in innovation, participants did not focus the discussion on the quality of medical interventions (i.e. the technical aspects of treating critically ill patients with organ dysfunction) provided. The sentiment expressed was that the quality of medical interventions received in Alberta ICUs is high. This finding may reflect that the public has implicit trust in the quality of medical care received [18].

While quality of medical care was not a focus, participants did heavily focus on communication, provider well-being and engagement as important themes to consider when improving the quality of critical care. Communication was a key focus for quality improvement during the café as previously reported in the literature [19]. Within the ICU, communication can be challenging due to the nature and severity of illness. ICU settings are characterized by uncertainty and place considerable strain on the family members, as most patients are unable to articulate for themselves [20, 21]. In support of the communication theme, participants spoke about the important role family members’ play as the surrogate voice of their loved one. Family members must communicate the patient’s wishes in terms of what care they would want to receive in their critical state [21–24]. To effectively act as the surrogate voice of the patient, family members must engage in conversations with their loved ones in order to understand their wishes for care.

The timing of such conversations is particularly relevant in critically ill patients who are frequently incapacitated due to the severity of their illness. As such, participants expressed the importance of having conversations with loved ones prior to an ICU admission. The literature indicates that family members’ who engaged in prior conversations or are aware of advanced care plans or directives feel better prepared to act as a surrogate voice [24, 25] including playing a pivotal role in daily decision-making [18]. Additionally, a recent systematic review suggests that seriously ill patients who completed directives have care preferences that are sufficiently stable over time and changes in health status, thereby allowing patient-centered decision making to occur even when patients lose their capacity and preferences can’t be reassessed [26].

Decision-making for ICU patients is complex, encompassing multiple components [27–29]. The volume of information families receive can be overwhelming [30, 31]; yet, healthcare providers need to elicit the patient’s values and treatment preferences early following ICU admission [25] and recognize these preferences may evolve over time or with changes in health status [26]. In addition, clinicians have reported several barriers to goals of care discussions with critically ill patients. Barriers include family members’ or patients’ difficulty accepting a poor prognosis, family members or patients’ difficulty understanding the limitations and complications of life-sustaining treatments, disagreement among family members about goals of care, and patients’ incapacity to make goals of care decisions [32]. It has also been suggested that healthcare providers may inadequately discuss goals of care with patients and families, adding to feelings of uncertainty and anxiety [33]. To address such deficiencies in communication, the Canadian Association of Research at the End of Life Network (CARENET) focuses its research on developing initiatives and tools to improve the quality of communication and decision-making between seriously ill patients, their families and health professionals (http://thecarenet.ca). Echoing the importance of such research, Café participants expressed that it would be beneficial to have access to decision tools that are designed to aid patients, families and providers in effective communication around understanding patient prognosis and options for care.

In recognizing the stressful environment encountered by patients and families during an ICU admission, participants were cognizant of the potential stress endured by ICU providers. The causes of high-levels of provider stress are thought to be multi-factorial, including long work hours and fatigue which can lead to impaired decision making and subsequent guilt and anxiety [34–36]. Decision-making around end of life care presents increased stress around both moral and ethical decision-making for the ‘right’ time and way to carry out comfort care [37, 38]. Café participants voiced concern that providers may not be given adequate opportunities to debrief and address causes of provider stress or suffering and that the network of support, both within the unit and outside of work, is likely limited. Although studies have explored the presence of and the causes for provider distress [39–42], little research has been conducted on interventions [43] designed to reduce distress [44]. Developing and implementing mechanisms to reduce high levels of provider stress was recognized as an important opportunity for improvement.

Public engagement around health care was a key theme during the café. Participants expressed a desire to be engaged through a variety of formats, with public forums being the most supported. Participants reported being grateful for being given the opportunity to engage in the café, given many felt current engagement opportunities are limited. Public forums, such as the Café Scientifique, can bring the public and experts together to engage in collaborative learning and multi-stakeholder priority setting, as well as provide an opportunity to share stories with others who have a similar lived experience. These findings highlight the importance of engaging the public in forums that allows the depth of the conversation to illicit understanding of public opinions beyond what can be accomplished with standardized questionnaires. While methodological summaries of the Café Scientifique method have been published [45, 46] to the best of our knowledge only one author has published summative findings from two CIHR Café Scientifiques held in Vancouver, B.C. focused on oral sex, oral cancer, and the HPV infection [47]. While Reimer-Kirkham and colleagues reported that the café method was limited in knowledge translation (e.g., disseminating scientific findings to the public), our experience suggested that the café method was effective for soliciting public opinions on how to improve the care. As such, the use of Café Scientifiques as a mode of public engagement is likely underutilized and represents an opportunity for many areas of medicine and research innovation.

There are three limitations to consider when considering our study findings. First, planning for a public event has various important considerations, such as appropriateness and accessibility of venue for the target population. It is possible that the time of the event or the location may have restricted members of the public from attending. However, the results of our quantitative feedback survey indicate overwhelming satisfaction with the Café, including the venue. Second, we did not collect demographic data from participants. Public forums are not a typical method for data collection, and as such we focused our feedback survey on participant satisfaction. Basic demographic information would identify ways to modify recruitment strategies to increase engagement and ensure all target stakeholders are adequately represented. Third, transferability of our findings should be assessed based on the understanding that participants were all from one geographic region within Alberta. Despite this, the study has a number of strengths. Coding and theme development were conducted individually and then as a group and included an experienced qualitative researcher. Coding was done immediately after transcription to ensure that observations made during the Café remained fresh in the researchers’ minds. Finally, the findings of the study align with previous research, increasing trustworthiness of the results.

The authors have reflected on the process of planning and delivering a Café Scientifique and offer the following recommendations for improving on such an event: (1) Ensure diversity in panel members speaking (e.g., various clinical and decision-making roles as well as patients and families); (2) Facilitate discussion between presentations as opposed to holding discussion to the end of all presentations; (3) Increase public awareness of event through all means possible (e.g., traditional news media; social media); (4) Consider hosting multiple events in various venues to increase reach; and (5) Consider planning a Café Scientifique in conjunction with other well-known public events and festivals.

## Conclusion

Given that care in the ICU is resource intensive [3, 48] and most often an unexpected occurrence for the patients and families, it is important to understand what aspects of care are priorities to patients and families, in addition to the providers who work in this field. We found the public focused their priorities for improvement on the way care is delivered (i.e. non-technical aspects of medical interventions), highlighting the need for patient and family centered care in the ICU. We found that a Café Scientifique was a valuable forum to allow the public to contribute to setting priority areas for quality improvement. To the best of our knowledge this has not been previously reported. In fact, the findings of our café have been presented to a provincial committee that has committed to work with ICU units across Alberta to support initiatives focused on enhancing communication, provider well-being, and engagement. Although participants chose not to focus on specific suggestions for improving medical interventions, our findings do highlight three aspects of care that participants felt are essential to the provision of high quality critical care. Engaging the public in forums provides a platform for the public to share their lived experience in an ICU, allowing other participants to gain an understanding of this unique care environment. As such, health care organizations, governments, research funding organizations should provide more opportunities for the public to engage in meaningful conversations about how to best improve healthcare. The Café Scientifique is one method to be considered.

## Supporting Information

S1 Appendix Participant Survey. (DOCX)

S2 Appendix Qualitative Coding Scheme. (DOCX)

S3 Appendix Feedback Survey Data. (XLSX)
