# Supplementary material for: Engaging the Public to Identify Opportunities to Improve Critical Care: A Qualitative Analysis of an Open Community Forum
Source: PLoS One. 2015 Nov 18;10(11):e0143088. doi: 10.1371/journal.pone.0143088 (PMC4651489; doi:10.1371/journal.pone.0143088)
Supplement: S1 Appendix — (DOCX) [file pone.0143088.s001.docx]

**Appendix 1: Participant Survey**

Your feedback is important to us, so we can continue to improve future public engagement events. Please take five minutes of your time to fill out this survey and return it to the **Registration Desk**.

1. How did you hear about the “How would you improve life-saving care in Alberta” event? (please indicate all that apply)

Event Poster Twitter Eventbrite

E-mail Colleague Electronic Bulletin Board

Facebook Faculty of Medicine Website

University of Calgary Website

Other (please specify): ________________________

1. How satisfied were you with the exploration of the event’s theme, which focused on improving the care of critically ill patients by identifying priorities for research and innovation in critical care medicine?

| **Very Satisfied** | **Somewhat Satisfied** | **Neutral** | **Somewhat Dissatisfied** | **Very Dissatisfied** |
| --- | --- | --- | --- | --- |
|  |  |  |  |  |

1. How satisfied were you with the relevance of the discussion given by the panellists?

| **Very Satisfied** | **Somewhat Satisfied** | **Neutral** | **Somewhat Dissatisfied** | **Very Dissatisfied** |
| --- | --- | --- | --- | --- |
|  |  |  |  |  |

1. Overall, how satisfied were you with the following aspects of the event?

|  | **Very Satisfied** | **Somewhat Satisfied** | **Neutral** | **Somewhat Dissatisfied** | **Very Dissatisfied** |
| --- | --- | --- | --- | --- | --- |
| **Scheduling & timing:** |  |  |  |  |  |
| **Facility & venue:** |  |  |  |  |  |
| **Food & beverage:** |  |  |  |  |  |

1. What do you think are the most important ways we can improve life-saving care in Alberta?

_______________________________________________________
 _______________________________________________________
 _______________________________________________________

1. How should efforts be prioritized to improve life-saving care?

_____________________________________________________
 _______________________________________________________
 _______________________________________________________

1. What do you think the priorities for improving life-saving care should be?

_______________________________________________________
 _______________________________________________________
 _______________________________________________________

1. What role should the public play in improving life-saving care?

_______________________________________________________
 _______________________________________________________
 _______________________________________________________

1. What did you like best about the event?

_______________________________________________________
 _______________________________________________________
 _______________________________________________________

1. What would you like to see done differently at the next critical care public engagement event?

_______________________________________________________
 _______________________________________________________
 _______________________________________________________

1. How likely are you to attend future critical care public engagement events?

| **Very Satisfied** | **Somewhat Satisfied** | **Neutral** | **Somewhat Dissatisfied** | **Very Dissatisfied** |
| --- | --- | --- | --- | --- |
|  |  |  |  |  |

Thank you for your valuable feedback. Please return this survey to the **Registration Desk**. If you would like to provide additional feedback, please send an email to [jamboyd@ucalgary.ca](mailto:jamboyd@ucalgary.ca).
