# Supplementary material for: Engaging the Public to Identify Opportunities to Improve Critical Care: A Qualitative Analysis of an Open Community Forum
Source: PLoS One. 2015 Nov 18;10(11):e0143088. doi: 10.1371/journal.pone.0143088 (PMC4651489; doi:10.1371/journal.pone.0143088)
Supplement: S2 Appendix — (DOCX) [file pone.0143088.s002.docx]

**Appendix 2: Qualitative Coding Scheme**

| **Exemplar Quote** | **Coding Scheme** | **Themes** |
| --- | --- | --- |
| “I think that just what was great about my experience with my mom is that the doctors, and the nurses especially, were just so, so beautiful and so, so caring and it was just really, really tough. And actually mom, mom had already passed away when my brother and my sister and the family came to see her. And my son, he came up to see her and she was just on breathing tubes. He didn’t recognize her at all. It was just a really, at that time, it was just a sad, sad situation for me but the nurses and the doctors really did a good job of letting me know that you know mom was very, very well cared for. Thank you.” | End-of-life care | Communication |
| “At least give them the information so they can make an advised decision on where they're going to go and how they're going to make those decisions for their families.“ | End-of-life communication |  |
| “We need to… help people understand the decisions that need to be made at the end-of-life. Should I be resuscitated? Should I go to the ICU? What’s this mechanical ventilation thing all about?” | Decision-making |  |
| “I was told when I came out of the ICU, I was still in the hospital for another 3 weeks after that recovering, and they kept wondering what happened, what happened. I was told over and over again ‘you don’t want to know, you don’t want to know”’. I said “really? I do want to know. It’s 10 days of my life what happened to me? Where was I? What went on?” My dad took a journal, so thankfully we had a record and then it was my choice to look at it or to not… I’m really grateful for my dad that he did that and I looked at it once, I probably won't ever look at it again, but it really, there was a big chunk of time of what was I doing.” | Family journal |  |
| “At that time when I went to see her she had the tubes in her throat and she couldn’t speak so we kind of wrote back and forth on an envelope. And then she kept writing over and over again ‘I want to be resuscitated. Please make sure you talk to the doctor and tell the doctor I want to be resuscitated.’” | Difficulty of communicating with loved one |  |
| “There has to be some conversation about as to what they would be comfortable in discussing, what you guys are comfortable in accepting from them. Do you actually talk about this particular thing? Do we need to talk about this particular thing again? It’s very distressing for families I think when you’re revisiting things over and over and over and over again.” | Pressure of being the patients voice |  |
| “At that time when I went to see her she had the tubes in her throat and she couldn’t speak so we kind of wrote back and forth on an envelope. And then she kept writing over and over again “I want to be resuscitated. Please make sure you talk to the doctor and tell the doctor I want to be resuscitated.” So I made sure that that’s exactly what I would do.” | Family as patient voice |  |
| “We just started to do things ourselves and it was nice that they let us do some things, showing confidence and respecting you.” | Trust |  |
| “It means a lot to family when they, the nurses, keep calling the patient by name and tell them what they are going to do even if he’s in a coma. It was the personalization that this was my husband.” | Relationships |  |
| “We need to see the support tools to help people understand the decisions that need to be made at the end-of-life. Should I be resuscitated? Should I go to the ICU? What’s this mechanical ventilation thing all about?” | Decision-tools for patients and/or families |  |
| “Way too late when people hit the ICU to be having these conversations. Way, way, way too late, first of all.” | Timing of conversations |  |
| “It’s too difficult and it’s too late when you’re in the ICU trying to make those decisions.” | Timing of decisions |  |
| “We then go to the chart and look at what their goals of care are and they disagree 2/3 of the time. Or said differently only 1/3 of the time to do their elicited preferences align with what’s documented on the chart and the majority of that disagreement is because people say ‘keep me comfortable" but they're a full code on the chart.‘” | Goals of care |  |
| “We're promoting the idea to start normalizing the conversation, clarifying values, and in general, overall what your goals in life and, you know, what’s worth living for, what are you looking for? So that’s not what we’re, we're not asking the family docs to make decisions about CPR. We're asking them to help prepare that person or that patient so that when they enter the acute-care system they are ready in the moment to make a decision with a doctor about...” | Advanced care planning |  |
| “I sometimes don’t feel… that the physicians are doing the best job of explaining the true story of what this treatment is going to look like and what the quality of life of that patient will be at the end. “ | Physicians not the best discipline for communication |  |
| “Asking [family doctors] to help prepare that person or that patient so that when they enter the acute-care system they are ready in the moment to make a decision.” | Conversations needed prior to illness |  |
| “So the sickest patients in the hospital come to the intensive care unit. There’s very little time for relationship between the care providers and the patient… because most of the patients are unconscious due to medications with tubes in their throats and tubes in their orifices or places and so they don’t interact with their care providers as much as families do. “ | Severity of illness limits communication |  |
| “Vicarious traumatization, where we vicariously live the trauma of our patients and loved ones and that causes us to suffer. “ | Staff stress | Provider Well-being |
| “Moral distress, that’s the stress that comes from, you know, offering or doing things that you morally are opposed to, that you don’t think are in the patient’s best interest. And again, research shows that for most of us that comes at that end-of-life.” | Moral distress |  |
| “So we have to be emotionally alive and able to have those conversations. But if we're suffering from vicarious trauma, if we're suffering from moral distress, we won't be very good at that” | Staff burden |  |
| “We know that suffering happens in ICU… how do we address the suffering of ourselves as healthcare providers caring for patients in often the worst times in their life?” | Emotional burden |  |
| “How do we address suffering of our patients… in often the worst times in their life?” | Addressing suffering of the patient |  |
| “Burnout or fatigue, you know, because we are giving, giving, giving and we don’t have processes in our workplace to fill our souls back up to enable us to keep giving.“ | Stress fatigue or burn-out |  |
| “You don’t want a depressed, vacant, distant healthcare provider to engage with you around the serious conversations about the care of your loved one.” | Depressed, vacant, distant provider |  |
| “Even though we care very deeply I hope we care very deeply for each other in the ICU as staff.” | Staff recovery after tragedy |  |
| “But I do wonder about the healthcare providers because you know when your loved one dies and eventually they leave the room presumably another patient could come in with another illness and to what extent do the healthcare providers get together and talk about it? What kind of caring do they have? What kind of an opportunity, because they [go] through some grief too.” | Lack of ability to debrief |  |
| “Just kudos to everybody here for actually getting involved in this and bringing it to the forefront. Very important.” | Grateful | Engagement |
| “First of all, thank you for having this forum tonight, this is fantastic. I appreciate the opportunity to be able to come and to speak and to share my thoughts.” | Appreciation |  |
| “Quite honestly I had the opportunity to share... my story in October with the [organization name] and I was humbled to be able to do it and I think it had an impact.” | Desire to be heard |  |
| “I always wondered about what would really happen if, because I've only got my story, what would happen if we learned the stories of lots of other patients and families.” | Power of Storytelling |  |
| “From a patient/family point of view, the key priorities are around quality and quality care relates a lot to relationships and trust and communication. Oddly enough they don’t say things like ‘you know I want the most expensive technology built to transplant my heart when I’m, you know at the end.’ Maybe, you know, that’s important but relative to relationships and communication and trust, it’s not as important. “ | Quality of care is more than technology |  |
| “I’m not convinced that surveys work at all. I think that you need to take the time and listen to stories.” | Keep talking |  |
| “The public needs to be informed and participate in the setting of care limits and boundaries in the ICU.” | Engage the public |  |
| “More conversations like this one needed. Thank you!” | More opportunities |  |
